# Corneal Cross-Linking Window Absorption (CXL-WA) as an Adjuvant Therapy in the Management of* Aspergillus niger* Keratitis

**DOI:** 10.1155/2018/4856019

**Published:** 2018-12-10

**Authors:** Pietro Rosetta, Emanuela F. Legrottaglie, Luca Pagano, Paolo Vinciguerra

**Affiliations:** Humanitas Clinical and Research Center, Via Manzoni 56, Rozzano, Milano, Italy

## Abstract

**Purpose:**

To evaluate the effectiveness of corneal cross-linking window absorption (CXL-WA) as an adjuvant therapy for* Aspergillus* keratitis.

**Methods:**

A 90-year-old male came to our clinic complaining of hyperemic conjunctivitis and progressive visual loss in the right eye. Slit-lamp examination showed keratic precipitates, severe corneal opacity, and stromal edema. Corneal scraping culture was positive for* Aspergillus niger*. Because the clinical condition did not sufficiently improve with antifungal therapy, the patient underwent CXL-WA as an adjuvant therapy.

**Results:**

During the first week after treatment, the Tyndall effect, corneal edema, and signs of ocular inflammation progressively lessened. At the third postoperative month, the cornea was stable without signs of fungal keratitis. However, after this period, a descemetocele appeared in the cornea (2 × 2 mm in diameter), so the patient underwent a corneal penetrating keratoplasty. Histological evaluation of the removed corneal tissue revealed the presence of hyphae and fungal infection.

**Conclusions:**

We reported a case of in vivo CXL-WA used as an adjuvant therapy for deep stromal* Aspergillus* keratitis. CXL did not completely eradicate the fungal infection which caused perforation 4 months after treatment and it still cannot be considered a definitive solution to mycotic keratitis, which maintains a poor long-term prognosis.

## 1. Case Report

A 90-year old Caucasian man came to our department in September 2017 for a routine check-up because of persistent untreated conjunctivitis in his right eye. The patient presented with hypertension treated with amlodipine and furosemide. The ocular history was positive for bilateral primary open angle glaucoma treated with Timolol 0.5%. The patient underwent ectropion surgery five years earlier (right lower lid lateral tarsal strip), bilateral cataract surgery with IOL implantation, and a later YAG-laser capsulotomy. On his left eye, an aponeurotic ptosis was present.

The clinical examination showed a best-corrected distance visual acuity (BCDVA) of 0.7 logMAR (20/100) in the right eye. Intraocular pressure was 20 mmHg in his right eye and 15 mmHg in the left eye. A slit-lamp examination showed conjunctival hyperemia and inferior brownish non-granulomatous endothelial precipitates. The corneal fluorescein staining was negative. The posterior segment did not show any pathological findings.

A viral etiology was suspected and, given that PCR analysis is not available in our clinic, an empirical treatment was started with Ganciclovir gel 1.5 mg/g, 3 times a day. Fourteen days later, the patient presented with a worsening of the clinical condition: BCDVA was hand movement, and he had a corneal paracentral ulcer (1 mm in diameter) with fluorescein staining. The ulceration was surrounded by a mild, localized corneal edema. The Tyndall effect was positive and the precipitates were more numerous.

A corneal scraping was then performed, and the clinical manifestation was highly suspected to be a fungal keratitis. Therapy was modified with 2 mg/ml topical voriconazole 1% 4/die and moxifloxacin 0.3% 6/die.

**Figure 1:**
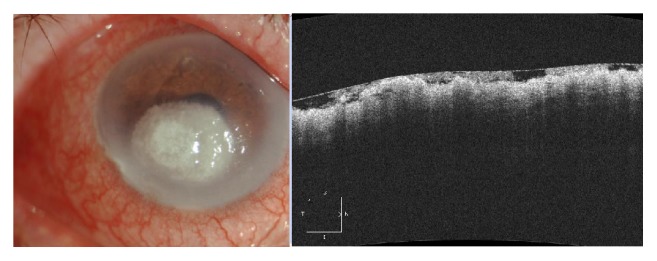
Anterior segment photography and corresponding AS-OCT image of the preoperative condition. It shows a round 4 × 4 mm corneal ulcer reaching the deep stroma, associated with massive melting surrounding the edema and perikeratic injection.

The cultural test on Sabouraud agar gave a diagnosis of* Aspergillus niger* infection. Despite the therapy modification, the corneal situation continued to worsen, with a perikeratic injection and increased size of the round corneal ulcer (4 mm in diameter) involving the optical zone and reaching the deep stroma (Figure 1) with massive melting and surrounding edema. AS-OCT images were not able to detect the effective pachymetry of the residual stroma because of the corneal melting.

The literature has reported promising results with CXL as adjunct therapy for fungal keratitis. Because the clinical condition became worse with antifungal therapy, and since we obtained* A. niger* positivity, we planned a CXL-WA [1] 7 days after corneal scraping. Informed consent was collected from the patient.

## 2. Surgical Procedure

Thirty minutes before the surgery, pain medication was administered.

The CXL-WA [1] treatment was performed under topical anesthesia with oxybuprocaine hydrochloride 0.2% and lidocaine 4%, administered 5 minutes before surgery.

The procedure was conducted in sterile surgical conditions. The patient was draped, the ocular surface was rinsed with sterile physiologic balanced salt solution, and a lid speculum was applied.

A hypo-osmolar 0.1% riboflavin solution (RICROLIN TE Sooft, Italy) was instilled for 30 minutes before irradiation, to obtain stromal swelling. The cornea was exposed to ultraviolet light A with the UV-X System (Peschke Meditrade GmbH, Huenenberg, Switzerland), which emits light at a wavelength of l370 ± 5 nm and an irradiance of 3 mW/cm^2^ or 5.4 J/cm^2^. Exposure lasted for 30 minutes, during which time riboflavin solution was applied 6 times, every 5 minutes. After surgery, the patient received levofloxacin drops (Oftaquix; Tubilux Pharma, Pomezia, Rome, Italy). Follow-up visits entailed photo documentation with and without fluorescein, AS-OCT (Cirrus, Karl Zeiss OCT, Germany), and analysis of the anterior chamber with Pentacam (Oculus Optikgerate GmbH, Wetzlar, Germany). Before treatment therapy was 2 mg/ml topical Voriconazole 1% 4/die and Moxifloxacin 0.3% 6/die. The first day after CXL-WA, therapy was modified by adding Betamethasone 0.1% + Naphazoline 0.15% + Tetracycline 1% (Alfaflor®) eye drops 3/die and Iodopovidone 0.6% eye drops 3/die, to reduce the inflammation and to prevent bacterial contaminations; moxifloxacin 0.3% was stopped.

**Figure 2:**
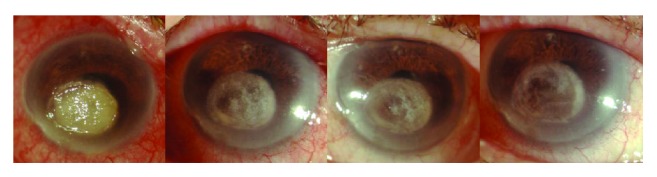
Anterior segment photography follow-up after CXL-WA: 1 day, 1 week, 1 month, and 2 months showing initial apparent stability of the clinical condition.

During the postoperative follow-up, from 1 week until the second month, the patient was reevaluated and showed a progressive reduction in the margins of the corneal opacity which was documented with anterior segment photography and AS-OCT (Figure 2).

One month after CXL-WA, no signs of keratitis reactivation were present: we documented a reduction in the conjunctival hyperemia and chemosis and a complete corneal reepithelialization, as well as an improvement in corneal transparency (Figure 2).

Until the third month after treatment, the patient underwent a weekly follow-up that confirmed corneal stability. AS-OCT and Scheimpflug images showed improvement in corneal transparency, despite a significant corneal thinning (thinnest point was 150 *μ*m).

**Figure 3:**
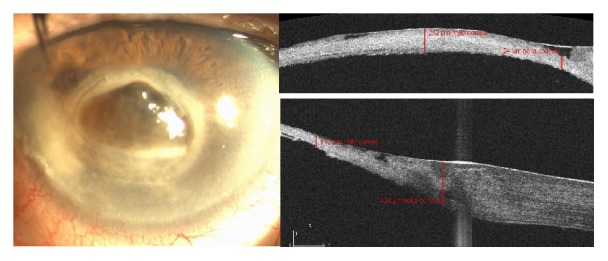
Anterior segment photography and AS-OCT showing a central 2x2 mm descemetocele with a thinning of the cornea (232 *μ*m centrally and 116 *μ*m peripherally).

In the fourth month after CXL-WA, the patient showed a 2 × 2 mm central descemetocele with a thinning of the cornea (232 *μ*m centrally and 116 *μ*m peripherally) (Figure 3).

**Figure 4:**
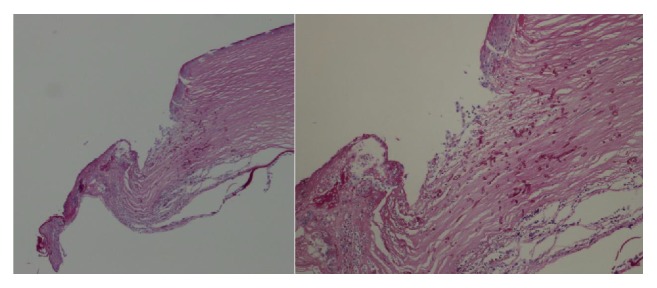
PAS positive section showing the focal erosion of the epithelium with a full thickness mixed inflammatory infiltrate, including lymphocytes, histiocytes, and granulocytes. Note the presence, in this context, of numerous branching hyphae which are highly suggestive of fungal keratitis.

In order to prevent a corneal perforation, we performed* a chaud *corneal penetrating keratoplasty. The histological evaluation of the removed corneal tissue revealed the presence of numerous branching hyphae and fungal infection (Figure 4).

## 3. Discussion and Conclusion

Fungal keratitis is caused mainly by two types of pathogens: yeast and mold. Yeast infection is often seen among patients with ocular diseases [2], while infection with mold (often called filamentous fungi) is more common in patients who have undergone trauma to the eye, who have an imperfect ocular occlusion, or who wear corneal contact lenses; the incidence of mold infection is higher than that of yeast infection. Antifungal topical drugs are produced in limited categories, and often the low penetration in the eye of this kind of drug makes the treatment of fungal keratitis difficult; as a consequence, the treatment outcomes remain poor [3]. Perforation is not uncommon. Other risk factors for poor outcomes include large and deep ulcers. Adjuvant therapies for advanced ulcers may be warranted in order to inhibit corneal melting and prevent perforation [4].

CXL of corneal stromal collagen fibers, induced by ultraviolet light A and riboflavin (Vitamin B2), has been postulated to have several mechanisms of action which could aid in the management of infectious keratitis. CXL increases the stiffness and enhances the mechanical and biological stability of the stroma by promoting the formation of covalent bonds among collagen molecules in the cornea; this effect minimizes the collagen lysis caused by pathogenic microorganisms [5, 6]. However, a recent study showed that, at a microscopic level, CXL did not reduce inflammation (metalloproteinases MMP-9 and MMP-13) [7]. Another mechanism could be related to the antimicrobial effect of the cross-linking itself, which may produce, through free radical production, an interference in the microbial cell wall [8–12].

As previously described, we decided to use the CXL-WA technique that we first introduced in 2013 [1]. It is a protocol for the treatment of infectious keratitis with some differences from the original Dresden CXL protocol [13]. As reported in the literature, in fact, in the presence of compromised ocular surface defense, such as corneal infection, chronic inflammation may lead to persistent epithelial defects [14]. To reduce the risk of delayed epithelial healing in CXL-WA, the epithelium is not removed. The penetration of riboflavin is obtained through the epithelial defect overlying the ulcer and the absence of the tight junctions of the epithelium.

In this case study, we also performed a manual debridement of the superficial melting prior to CXL-WA. This helped clear out part of the superficial mycotic melting as well as allowing the cross-linking effect to directly reach the deep stromal layers. Another difference from the standard protocol is the riboflavin solution used. In the presence of an acute infection and corneal ulcer, it is not possible to determine which part of the residual pachymetry is attributable to the stroma and which is attributable to melting. For this reason, a hypo-osmolar riboflavin solution is recommended, to induce corneal swelling and to avoid the risk of the treating residual stromal bed too thin. The use of photoactivated riboflavin in the treatment of melting corneal ulcers was first proposed in 2000 [15], and the first clinical application of CXL in treating infectious keratitis was reported in 2008 [16].

Several case reports have shown the effectiveness of CXL: in the treatment of recalcitrant bacterial keratitis, in the improvement of the clinical symptoms, in the halting of the progressive melting, and the resolution of infectious treatment-resistant keratitis [16–20]. Austin et al. published a review in 2017 supporting the use of CXL in bacterial keratitis [21]; they also tried to assess the effectiveness in fungal keratitis, but they found less robust evidence to support it.

In vitro studies are controversial: the CXL procedure alone has not been shown to be effective against inactivate fungus, although another study showed that CXL plus amphotericin improved inhibition of fungal pathogens over amphotericin alone [22, 23]. Ozdemir found that PACK-CXL and PACK-CXL combined with voriconazole were more effective than voriconazole alone in reducing the number of colony-forming-units in* Fusarium* and* Candida* keratitis [24].

The results of the available clinical trials are also controversial. A previous trial studied the effectiveness of CXL versus antimicrobial treatment alone in patients with bacterial, fungal,* Acanthamoeba*, or mixed-origin keratitis [25]. Although this trial found a lower complication rate (perforation or recurrence) in the CXL group, it had multiple limitations, including inappropriate randomization, inclusion of patients with any kind of keratitis, and insufficient power [26].

Papaioannou et al. [27] conducted a systematic review and meta-analysis of 25 studies (2 randomized controlled trials, 13 case series, and 10 case reports) in which 175 eyes were treated with CXL and 35 control eyes received antibiotic therapy alone. CXL was usually employed as an adjuvant with the antimicrobial treatment rather than a standalone treatment. There was a high success rate in treating bacterial keratitis (defined as complete epithelialization and infiltrate resolution), whereas the efficacy on fungal cases was more limited [27].

A small randomized clinical trial investigating cross-linking as adjuvant therapy for deep fungal ulcers suggested that CXL could increase the rate of perforation in this condition [28]. However, Richoz et al. pointed out that it was prematurely stopped based upon 4 perforations, so sufficient conclusions could not be drawn on either the efficacy or lack of efficacy of cross-linking [29]. In addition to this, they also suggested that even the depth of the fungal infiltrates could play a role. After the accumulation of fungal organisms in the deepest corneal layers, CXL could cause a sudden release of foreign fungal antigen, inducing a short but marked host inflammatory response that might transiently enhance corneal melting [29]. Moreover, Vaipayee et al. [30] retrospectively reviewed patients with moderate mycotic keratitis. CXL on the day of presentation plus intensive topical antifungal therapy did not significantly differ from the antifungal therapy alone in terms of healing time, final best-corrected visual acuity, or complications.

Another retrospective analysis was done by Erdem [31], who analyzed 13 patients with mycotic keratitis. He found controversial results in* Aspergillus* and* Fusarium* keratitis, with half of the patients healing and half showing a progressive melting. He concluded that CXL treatment is effective only in patients with small and superficial ulcers.

Most of these studies display the same problem: it is difficult to have a homogenous and standardized pool of cases, due to the low incidence of this condition and the different stages of presentation. Despite this, and although there is not as much evidentiary support for using CXL to treat fungal keratitis, it is already used in conjunction with antifungals by some clinicians, in the hope that it might add some benefit, given the poor prognosis for fungal ulcers [21].

In summary, the literature shows no clear path to follow in advanced cases and, unfortunately, to date, the most likely scenario is still to end up with penetrating keratoplasty.

In this case study, CXL was performed as an attempt to halt the progression of the infection, and we can speculate that it helped stabilizing the cornea in the short to midterm (3 months). However, there were confounding factors that make the effects of the CXL less clear. In fact, after having performed the CXL, we modified the therapy by adding Dexamethasone; steroids are known to improve the symptoms but not the long-term outcomes. Further, because of the ongoing antifungal treatment, the initial clinical improvement could not be entirely attributed to CXL.

Nevertheless, CXL did not completely eradicate the fungal infection which caused perforation 4 months after treatment. This is confirmed by the presence of fungal hyphae in the corneal tissue analyzed during penetrating keratoplasty.

For all these reasons, we believe that CXL-WA cannot be considered the definitive solution to advanced mycotic keratitis, which still maintains a poor long-term prognosis.
